# HTS discovery of PARP1-HPF1 complex inhibitors in cancer

**DOI:** 10.1016/j.slasd.2023.10.003

**Published:** 2023-10-14

**Authors:** Timothy Kellett, Rida Noor, Qiong Zhou, Hector Esquer, Rita Sala, Petra Stojanovic, Johannes Rudolph, Karolin Luger, Daniel V. LaBarbera

**Affiliations:** a Department of Pharmaceutical Sciences, The Skaggs School of Pharmacy and Pharmaceutical Sciences, University of Colorado Anschutz Medical Campus (CU AMC), Aurora, CO, USA; b Department of Biochemistry, University of Colorado Boulder, Boulder, CO, USA; c The CU AMC Center for Drug Discovery, Aurora, CO, USA; d Howard Hughes Medical Institute, University of Colorado Boulder, Boulder, CO, USA; e The University of Colorado Cancer Center, Aurora, CO, USA

**Keywords:** PARP1/2, HPF1, HTS, Assay development, Robotic automation, Cancer, DNA damage response

## Abstract

PARP1/2 inhibitors (PARPi) are effective clinically used drugs for the treatment of cancers with BRCA deficiencies. PARPi have had limited success and applicability beyond BRCA deficient cancers, and their effect is diminished by resistance mechanisms. The recent discovery of Histone PARylation Factor (HPF1) and the role it plays in the PARylation reaction by forming a shared active site with PARP1 raises the possibility that novel inhibitors that target the PARP1–HPF1 complex can be identified. Herein we describe a simple and cost-effective high-throughput screening (HTS) method aimed at discovering inhibitors of the PARP1–HPF1 complex. Upon HTS validation, we first applied this method to screen a small PARP-focused library of compounds and then scale up our approach using robotic automation to conduct a pilot screen of 10,000 compounds and validating >100 hits. This work demonstrates for the first time the capacity to discover potent inhibitors of the PARP1-HPF1 complex, which may have utility as probes to better understand the DNA damage response and as therapeutics for cancer.

## 1. Introduction

Poly (ADP-ribose) polymerase 1 (PARP1) is a critical enzyme for the DNA damage response (DDR) as it serves as a first responder to single and double strand DNA breaks (SSB or DSB) involved in base excision repair, non-homologous end joining, and homologous recombination (HR) repair [1–3]. PARP1 catalyzes the transfer of negatively charged ADP-ribose from NAD^+^ to target proteins such as histones and PARP1 itself, a process termed PARylation. During DDR, PARylation of histones leads to chromatin relaxation, recruitment of proteins needed for DNA repair, and release of PARP1 from damaged DNA. Inhibitors of PARP1/2 (PARPi) became of interest due to synthetic lethality wherein tumor cells lacking BRCA1/2 function are especially sensitive to PARPi, leading to specifically targeted tumor cell death [4]. Currently, there are four different PARPi approved for clinical use (olaparib, rucaparib, niraparib, and talazoparib) that target the active site of PARP1. PARPi have proven a useful tool in combating recurrent ovarian cancer [5], and have more recently been approved for other indications such as prostate cancer, breast cancer, and pancreatic cancer. However, over 40 % of patients with mutant BRCA1/2 ovarian cancer fail to respond to PARPi, and many patients acquire resistance after repeated administration of PARPi [6]. Therefore, it’s prudent to explore alternative methods to target PARP, which may circumvent the mechanisms responsible for PARPi resistance [7].

Recently, Histone PARylation Factor 1 (HPF1) was shown to be a critical component of the PARylation reaction by forming a shared active site with both PARP1 and PARP2 [2–4,8–11]. HPF1 facilitates the PARylation of histones (vs. autoPARylation of PARP1 and PARP2), changes the reaction specificity from modification of Glu/Asp-residues to Ser-residues, and yields much shorter PAR chains. These findings suggest that more specific and higher potency inhibitors could be developed for the shared active site of the PARP1–HPF1 complex. This approach may be more specific than the current PARPi since the concentration of HPF1 is ~20 times lower than that of PARP [9], PARP1 is known to have many other roles in the nucleus that are unrelated to the DNA damage response [12], and DDR appears to be mediated by HPF1-directed PARylation of Ser-residues [11]. Towards this goal, we have developed and validated a PARP1–HPF1 complex assay for HTS. Further, we have optimized this assay to rapidly screen tens of thousands of compounds using robotic automation, which is expected to facilitate the discovery and development of a new class of inhibitors targeting the DDR in cancer.

## 2. Materials & methods

### 2.1. Reagents

The double-stranded p18mer* DNA oligonucleotide was obtained from IDT: p18mer: 5′-phosphate-5′-fluorescein-GGGTTGCGGCCGCTTGGG-3′. Wild-type human PARP1 [13], PARP2 [14], and HPF1 [10] were expressed and purified as described previously. Binding buffer was used for all FP assays (50 mM Tris–HCl (pH 8.0), 50 mM NaCl, 1 mM MgCl_2_, 0.1 mM EDTA, and 0.01 % IGEPAL). Olaparib and benzamide were obtained from Tocris and Sigma-Aldrich, respectively, and stock solutions were prepared in DMSO. Low flange black flat bottom polystyrene NBS microplates (#3575) and V-bottom shaped 96-well plates (#3898) were obtained from Corning. A commercial library of 470 plausible PARPi were obtained from OTAVA chemicals, Ltd for the primary screen and assay validation. All PARPi from the OTAVA library were prepared in DMSO at an initial concentration of 8 mM before being diluted further and an Opentrons OT-2 robot was used to prepare serial dilutions of the compounds in DMSO in a 96-well plate (Corning #3898).

### 2.2. HPF1-effect on PAR chain length

1 μM PARP1 was incubated with 1 μM p18mer DNA and was PARylated by addition of 200 μM NAD^+^ for 15 min, with or without the presence of 5 μM HPF1. Reactions were quenched by the addition of Laemmli buffer to 1x, boiled for 3 min, and then run on 4–20 % SDS-PAGE. Gels were stained for protein using Blazin’ Blue (GoldBio).

### 2.3. Screening of a PARP-directed library

An Opentrons OT-2 robot was used for all the pipetting steps as described. First, 20 μL of binding buffer was placed into each well of the 384-well assay plate. Next, 10 μL of the pre-formed complex of PARP1 - HPF1 - DNA or PARP2 – HPF1 – DNA (prepared in binding buffer) was added to all wells. Next, 1 μL of the inhibitors from the 12-point dilution were added to the 384-well plate and was incubated with the protein - DNA for 30 min at room temperature (RT). The plate was then transferred to BMG Labtech CLARIOstar plate reader equipped with a syringe injector. The injector was used to add 10 μL of NAD^+^ into all wells except for eight control wells. The FP signal was read (excitation at 482 nm (bandwidth 16 nm), dichroic filter at 496 nm, and emission at 530 nm (bandwidth 40 nm) from the top of the plate) in the same order and at the same frequency (1/s) 10 min following the initiation of PARylation by addition of NAD^+^. Final concentrations were 5 nM p18mer (fluorescein-labeled), 10 nM PARP1/2, 200 nM HPF1, 200 μM NAD^+^, and 0.09 μM – 200 μM inhibitor. IC_50_ values were calculated using a nonlinear regression analysis (sigmoidal dose-response fitting with variable slope) with the following equation: *Y* = Bottom + (Top – Bottom)/(1 + 10(LOGIC50 – *X*) · HILL SLOPE) using GraphPad Prism.

### 2.4. Pilot HTS of a 10,000 compound diversity set

A Revvity G3 Explorer collaborative robot equipped with a freezer containing the drug library, a Revvity Janus liquid handler, and a Revvity Envision was used to carry out the screen. A ChemBridge diversity library containing 10,000 compounds was used for screening. Two identical daughter plates (2 mM) were generated, one for HTS and one for cherry-picking hits identified from HTS. The drug library was maintained in a LiCONiC STX500-DF Deep Freezer at −20 °C with 80 % N_2_. Binding buffer (13 μL) with 0.04 % DMSO was added to columns 23 and 24 of 32 384-well plates (Corning 3575). Next, 24.5 μL binding buffer was added to all wells on the plates. Then, 0.5 μL of 2 mM stock solutions for each of the 10,000 compounds was added to separate wells, giving a final concentration of 20 μM, excluding columns 1, 2, 23, and 24. Afterwards, 12.5 μL premix (PARP1 – HPF1 – DNA, at 10 nM PARP1, 100 nM HPF1, and 5 nM FITC-labeled p18mer DNA) was added to all wells. Next, 12.5 μL of 2 mM NAD^+^ was added to each well. Plates were incubated for 15 min at RT and the FP (excitation at 482 nm (bandwidth 16 nm), dichroic filter at 496 nm, and emission at 530 nm (bandwidth 40 nm) was read using a Revvity Envision. Columns 1 and 2 were used for the negative control of DMSO, and columns 23 and 24 did not have NAD^+^ added, serving as the positive control. Data for screened compounds was normalized between 100 % (positive control, rows 23 and 24) and 0 % (negative control, rows 1 and 2). The signal for the compounds was normalized to generate enzyme activity for each compound. Hits were defined using Standard Deviation thresholds based on the average response of the screened compounds over all plates.

Dose response curves for the hits were performed using the same Revvity robot system as for the initial screen. Binding buffer (13 μL) with 0.04 % DMSO was added to columns 23 and 24 of 32 different Corning (Cat. No. 3575) plates. Next, 25 μL binding buffer was added to all wells on the plates. Cherry-picked compound plates generated from the compound source used in the initial HTS were used to add 1 compound per column from 20 to 160 nM in duplicate with 2x serial dilution using a Revvity FlexDrop iQ instrument. A 12.5 μL premix (PARP1 – HPF1 – DNA, as above) was added to all wells. Afterwards, 12.5 μL of 2 mM NAD^+^ was added to each well and plates were incubated for 15 min at RT. The FP signal was read as above, and data for screened compounds was normalized between 100 % (positive control, rows 23 and 24) and 0 % (negative control, rows 1 and 2). Dose response curves were plotted and fit using GraphPad Prism with a non-linear regression analysis as described above.

### 2.5. Cell culture

SUM149PT cell line was purchased from the Anschutz Cell Technologies Shared Resource and short-tandem repeat (STR)-profiled and mycoplasma-tested before use. Cells were maintained in F12/Glutamax (Gibco) medium supplemented with 5 % fetal bovine serum (FBS), 10 mM HEPES, 1 μg/mL hydrocortisone and 5 μg/mL insulin and kept in a humidified incubator at 37 °C and 5 % CO_2_.

## 3. PAR immunofluorescence

18,000 SUM149PT cells were seeded into a 96 well PhenoPlate (Revvity) and allowed to adhere overnight. Cells were then treated with the selected hits at a concentration of 10 or 12.5 μM and 0.01 % final concentration of MMS for 5 h. The medium was then aspirated, and cells were fixed with 4 % paraformaldehyde for 15 min at room temperature (RT). After washing twice with PBS, cells were incubated with 0.3 % Triton X-100 for 15 min followed by another washing step. To decrease unspecific binding of the antibodies, cells were blocked in 5 % milk for 30 min at RT. Afterwards, cells were incubated with the anti-PAR primary antibody at a 1:1000 dilution for 2 h at RT. Cells were washed again and incubated with a goat anti-mouse AlexaFluor 647 secondary antibody at a 1:500 dilution for 1 and 2 h at RT. Finally, cells were washed, stained with Hoechst 33342 (1:1000), and imaged using a 40x water objective on the Phenix Plus High-Content Screening (HCS) System (Revvity). Each bar graph is representative of two experiments, each with three replicates of whole-well analysis per condition. The reported data represents the average of each cell within the replicates, plotted with standard deviation error bars.

### 3.1. Cell line tumor organoid culture and cytotoxicity

SUM149PT cells were plated at 2000 cells/well into 96-well Clear Round Bottom Ultra-Low Attachment plates (Cat. No. 7007, Corning, Corning, NY) in complete growth medium. Cell assay plates were centrifuged at 1000 RPM for 15 min to promote cellular aggregation, then 25 μL of cold Growth-Factor Reduced (GFR) Matrigel (10 %) was added per well for a final concentration of 2 %. Tumor organoids were then allowed to grow for 72 h prior to drug treatments, then treated with olaparib and compound 4 for an additional 96 h before preparing organoids for cell viability assessment with CellTiter-Glo 3D (Cat. No. G9683, Promega, Madison, WI). Briefly, tumor organoids were transferred to 96-well CELLSTAR white solid bottom plates (Cat. No. 655083, Greiner Bio-One, Monroe, NC), 40 μL (1:1) of CellTiter-Glo 3D was added to each well and plates were then placed on an orbital shaker at 450 RPM for 45 min at room temperature. CellTiter-Glo 3D’s luminescence signal was assessed using the Envision microplate reader (Revvity, Waltham, MA) with a measurement time of 100 ms. The experiment was done with triplicate organoids per condition, and IC_50_.values were calculated as described above.

## 4. Results

### 4.1. HTS assay development targeting PARP1-HPF1

**Fig. 1.**
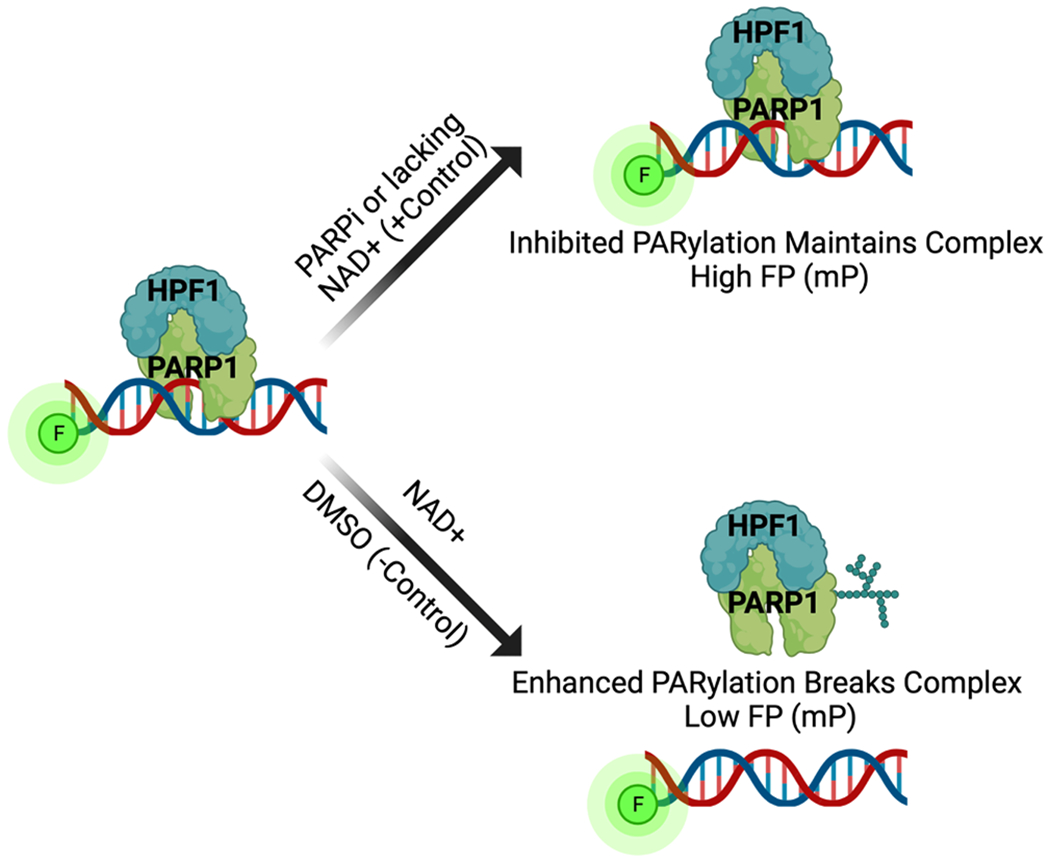
Scheme demonstrating the principle of the fluorescence polarization (FP) assay. In the presence of an inhibitor or lack of NAD+, the PARP1–HPF1–DNA complex is maintained, and a higher FP signal is observed (top arrow). In the absence of an inhibitor with NAD+, the pre-formed PARP1–HPF1–DNA complex becomes PARylated, leading to the dissociation of the DNA complex and a lower FP signal (bottom arrow).

**Fig. 2.**
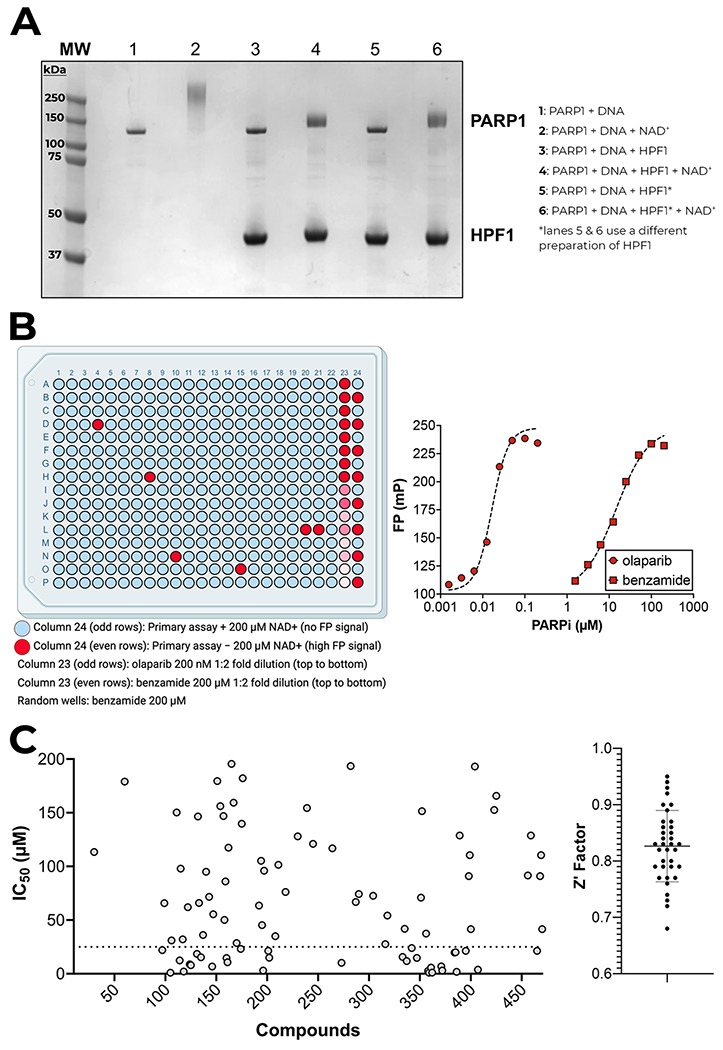
Development of Assay Method. (A) Demonstration of HPF1-effect on PAR chain length. PARP1 was incubated with p18mer DNA (lane 1) and was PARylated by addition of NAD^+^ for 15 min, leading to a smear of high MW PARP1 (lane 2). In lane 3, PARP1 was incubated with HPF1 without the addition of NAD+. In lane 4, upon addition of NAD^+^, PARylation of PARP1 is observed to yield much shorter chains. Lanes 5 and 6 show the same experiment as lanes 3 and 4, respectively, using a different preparation of HPF1. (B) Left: Heat map of HTS primary assay FP data in a 384-well plate. Negative controls and non-inhibitor samples are blue. Positive controls and inhibited samples are red. Column 24 (odd rows): Primary assay + 200 μM NAD+ (no FP signal), (even rows): Primary assay – 200 μM NAD+ (high FP signal). Column 23 (odd rows): olaparib 200 nM 1:2 fold dilution (top to bottom), (even rows): benzamide 200 μM 1:2 fold dilution (top to bottom). Six random wells were blind-spiked with 200 μM benzamide and demonstrates that the assay performs well throughout the plate. Right: Dose-response graphs for olaparib (IC_50_: 17 nM) and benzamide (IC_50_: 14 μM). (C) A primary screen was performed to determine the 25 most potent compounds using the PARP1–HPF1 complex. Data points >200 μM are not shown for graphical clarity. Compounds with IC_50_ < 25 μM (dotted line) were subjected to a second round of testing in triplicate.

We base our assay method on our previously described fluorescence polarization (FP) assay for measuring the activity of PARP1 [15]. Here we have optimized and validated its implementation for the PARP1–HPF1 complex using a HTS format [16]. We begin by forming a PARP1–HPF1–DNA complex wherein the DNA is fluorescently labeled (FITC-488) (Fig. 1). We validated the formation of the PARP1-HPF1-DNA complex by demonstrating that the addition of HPF1 to the PARP1-DNA complex perturbs the activity of PARP1, leading to much shorter PAR chains on PARP1 following PARylation as previously reported by us [17] and others [10,12]. (Fig. 2A). For the inhibition assay, the complex is incubated with potential inhibitors and the PARylation reaction is initiated by the addition of NAD^+^. PARylation leads to the disruption of the complex, which is revealed by a change in the FP signal (low FP) after 10 min of reaction time. Conversely, when inhibited, the PARP1–HPF1–DNA complex is maintained, providing a high FP signal (Fig. 1). We validated this assay for use in 384-well plates with a Z’ = 0.89 [18]. The assay unambiguously identifies randomly placed inhibitors or controls regardless of the well location (Fig. 2B). Additionally, each plate contains an 8-point titration of a positive control, either the PARPi olaparib (tight-binding, IC_50_ = 17 nM), benzamide (weak-binding, IC_50_ = 14 μM), or no NAD^+^ that yield highly reproducible measurements including drug potency and the ability to identify both tight- and weak-binding inhibitors (Fig. 2B).

### 4.2. Screening of a PARP-directed library

**Fig. 3.**
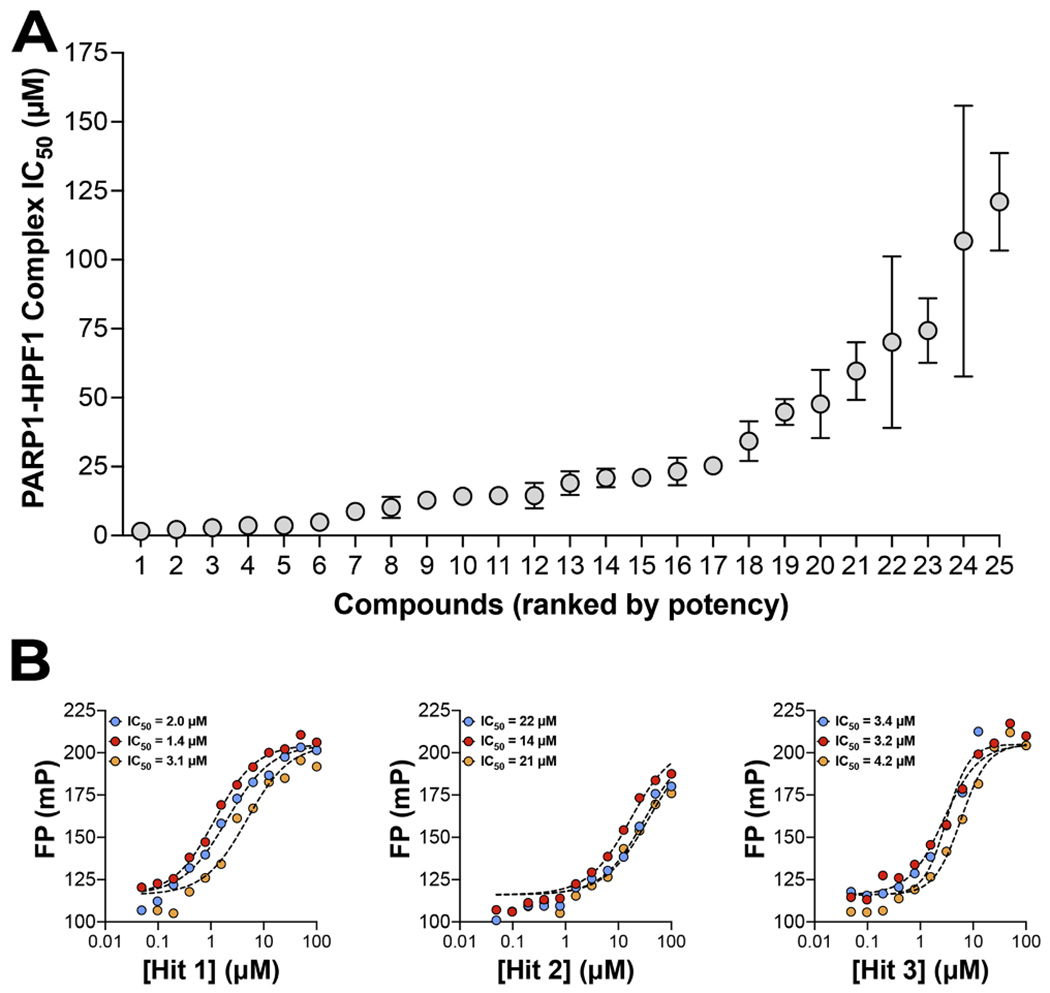
Confirmation of pilot screen hits. (A) The determined IC_50_ values of the 25 most potent hits, done in triplicate and plotted with mean ± SD. (B) Representative data for three different hit compounds, showing 3 replicate dose response curves per hit on the same graph, demonstrate the reproducibility of this assay.

Initially, to increase our chances of hit identification, we screened the PARP1–HPF1–DNA complex with a library of diverse, high quality, small molecule PARP-directed inhibitor library comprised of 470 compounds. All compounds were assayed in 12-point titrations (90 nM – 200 μM, 1:2 dilutions) using an Opentrons robot to assist with serial dilutions and sample additions. Control samples generated an average Z’ = 0.8 ± 0.06 and we identified 25 compounds with apparent potencies below 25 μM (Fig 2C and Supplementary Table 1). To validate these 25 most potent compounds, the compounds were re-tested in triplicate for the inhibition of PARP1–HPF1 complex using the same assay method. The average Z’ = 0.82 ± 0.02 in this re-screening (Fig. 3A and Supplementary Table 2) and representative IC_50_ curves further demonstrate that the HTS assay is highly reproducible and identifies compounds with potencies ranging from 1 to 25 μM (Fig. 3B).

### 4.3. HTS pilot screen of a 10,000 compound diversity set

**Fig. 4.**
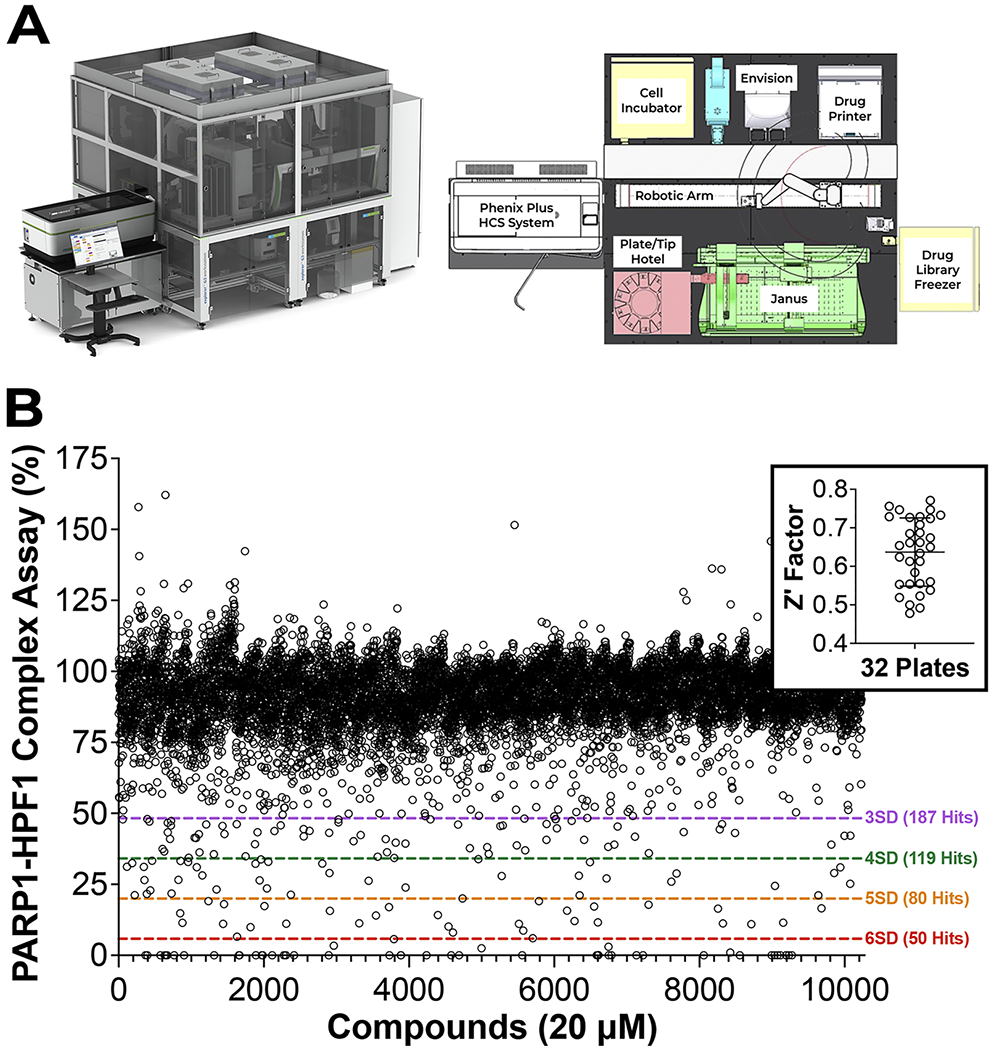
Screening and validation of a 10,000-compound screen. (A) A schematic of the automation platform used for the 10,000-compound diversity set HTS (B) HTS data plotted as a scatter plot with Z’ (inset). Standard deviation (SD) hit limits were used as indicated to determine the 187 most potent hit compounds.

**Fig. 5.**
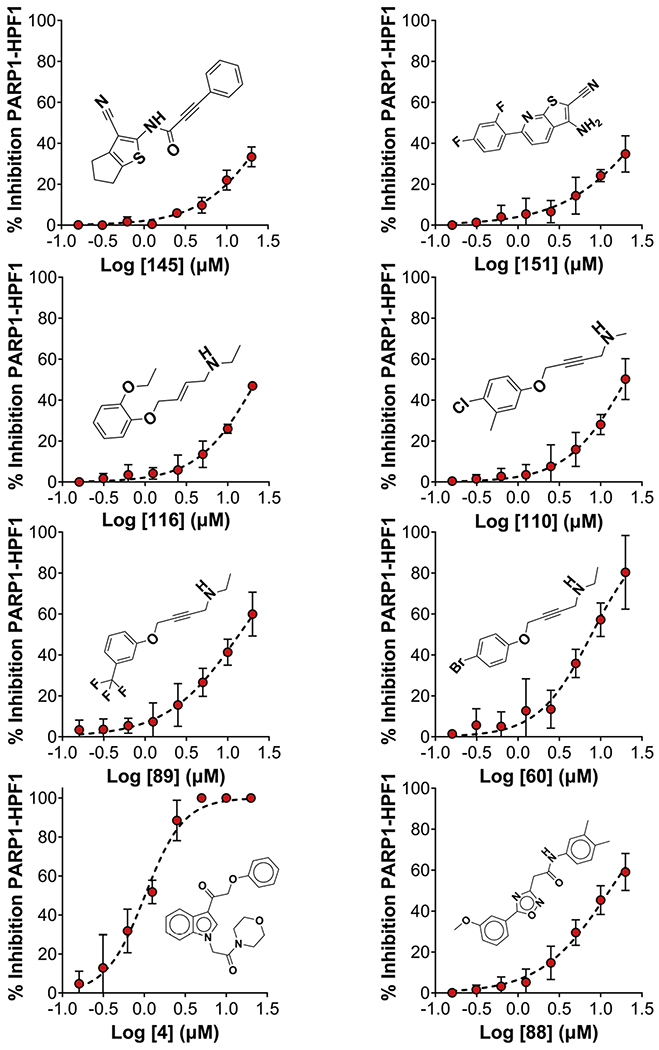
Representative dose-response graphs and structures including analogs of eight of the validated hits from the 10,000-compound HTS. The data was obtained from duplicate experiments with the mean ± SD.

We next adapted the HTS assay for robotic automation to screen a library of 10,000 compounds from a diversity library (ChemBridge) to discover novel compounds that specifically target the inhibition of the PARP1–HPF1. The library was designed by filtering out unwanted compounds known to provide false positive activity (aka PAINS) [19]. The diversity set is comprised of compounds with drug-like physicochemical properties and contain analogs to facilitate hit-to-lead validation and provide some structure activity relationships (SAR) directly from HTS. A Revvity G3 Explorer automation platform featuring a P-Flex robotic arm integrated with an Opera Phenix Plus, G3 Janus liquid handler, Envision multimode plate reader, and other equipment was used to carry out HTS in 384-well plates (Fig. 4A). The HTS Z’-factor over 32 plates had an average of 0.64 ± 0.09 and we set our hit limit to be 3SD from the mean signal indicating a 99.7 % confidence with 187 hits and an approximate 2 % hit rate (Fig. 4B). The 187 hits were validated using the primary HTS assay measuring the dose-response of hits. Notably, 166 out of 187 (89 %) hits were confirmed with dose dependent activity. Representative hit dose responses and structures are shown from the validated hits (Fig. 5). Notably, several of these compounds are analogs. Overall, HTS identified an array of different PARP1–HPF1 complex inhibitors, including several potent hits with IC_50_ values below 2 μM (Supplementary Table 3).

### 4.4. Hit validation in a cell-based assays

**Fig. 6.**
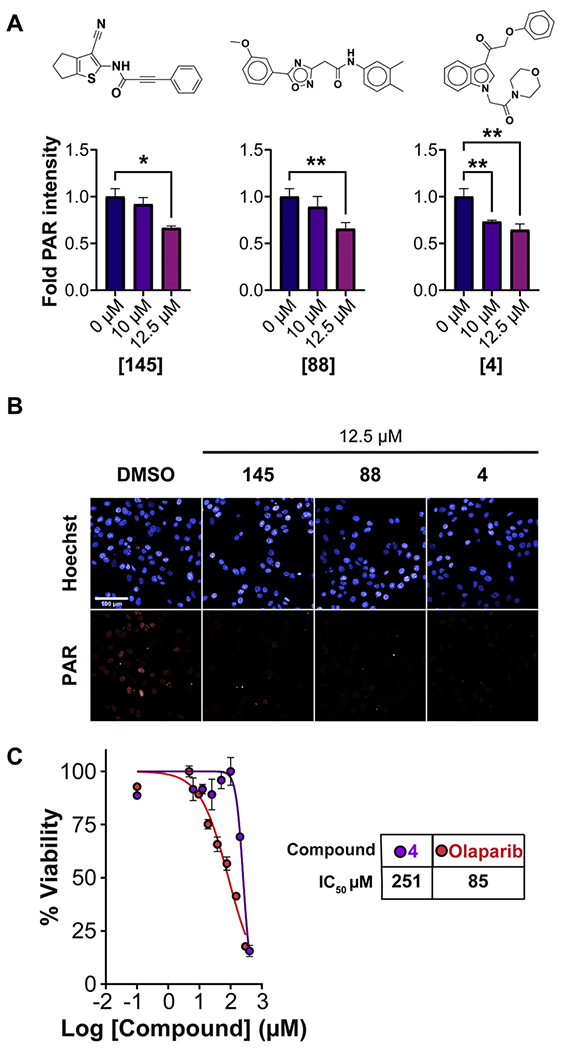
Hit validation in breast cancer-cell based assays. (A) Levels of PAR in the nucleus measured by immunofluorescence after incubation of SUM149PT cells for 5 h with 10 or 12.5 μM of the hits and 0.01 % final concentration of MMS. (B) Representative images of the PAR immunofluorescence of control and treated SUM149PT cells. Scale bar = 100 μm. (C) Dose-response curves for hit compound 4 and olaparib in SUM149PT 3D spheroids after 96-hour treatment, plotted with mean ± SD, along with calculated IC_50_ values for hit compound 4 and olaparib from the dose response curves.

It is well known that PARPi decrease PARylation within cell nuclei [20]. Thus, to validate HPF1-PARP1 complex inhibitors we measured PAR in the nucleus after treatment with hits, including 4, 60, 88, 89, 110, 116, 145, and 151. Cells were treated with 10 and 12.5 μM of the hits for 5 h, followed by PAR immunofluorescent staining and quantitation using high-content imaging and analysis. To potentiate DNA damage and subsequent activation of PARP1 and PARylation, 0.01 % final concentration of methyl methanesulfonate (MMS) was added to the media of control and treated cells. Of these hits, compounds 4, 88, and 145 showed a dose-dependent inhibition of PARylation (Fig. 6A–B). Hit compounds 145 and 88 decreased the amount of PAR in the nucleus at the higher dose tested (*p*<0.05), while 4 reduced PAR at all doses (*p*<0.001), correlating with its more potent activity in the FP assay. Finally, we confirmed the activity of the most potent hit, compound 4, in a tumor organoid cytotoxicity assay. After 96 h treatment, compound 4 inhibited tumor organoid viability with an IC_50_ of 251 μM, compared to 85 μM for the PARPi olaparib (Fig. 6C). These assays demonstrate utility as secondary assays to confirm hits identified from primary HTS.

## 5. Discussion

We and others have recently demonstrated that HPF1 significantly alters the enzymatic activity of PARP1 [2–4,8–11]. These changes include a quantitative switch in PARylation of Glu/Asp-residues to Ser-residues, a significant shift in PARylation from self (autoPARylation) towards histones (transPARylation), significantly shorter PAR chains, and an increase in “treadmilling”, the formation of free ADP-ribosylation (ADPr) instead of attached ADPr. These influential changes in PARP1 activity are orchestrated by HPF1 via a shared active site in the PARP1–HPF1 complex, wherein HPF1 provides a catalytic base to promote PARylation of Ser-residues, which in turn blocks the second ADPr binding site to prevent chain elongation. Furthermore, this unique interaction creates a binding groove for histone tails to promote trans-PARylation.

The significantly different active sites between free PARP1 and the PARP1–HPF1 complex that mediate all these changes in activity suggest that PARPi could be discovered and developed to target this shared active site. Additionally, PARP1–HPF1 directed inhibitors may have advantages over existing PARPi due to increased specificity that would likely alleviate any potential off-target effects. Our work describes a simple, reproducible, and cost-effective blueprint to discover novel PARP1–HPF1 complex inhibitors. Notably, pre-mixing optimized nM concentrations of protein and labeled DNA combined with robotic automation greatly reduces the number of steps in our workflow. Furthermore, utilizing an FP-based signal readout provides for a robust HTS assay. The 10,000-compound screen had a hit rate nearing 2 %, however, in a subsequent larger screen this could be reduced by increasing the empirical hit limit beyond 3SD from the mean as we show in Fig. 4.

In addition to the primary HTS screen, we also implemented a high throughput method for confirmation of the HTS hits via a dose-response assay. The use of the Revvity FlexDrop iQ allowed accurate dispensing of nL volumes of drug into the assay plates to produce accurate dose responses without the need of manual pipetting or serial dilution. Our high-throughput dose-response confirmation assay validated 166 out of 187 initial hits, streamlining the hit confirmation process. Follow up hit validation in a secondary cancer cell-based PARP-mediated PARylation assay effectively validated hits that have *in vitro* inhibitory activity against the HPF1-PARP1 DNA complex.

PARPi also inhibit auto-PARylation, which elicits cytotoxicity to BRCA mutant tumor cells. To further validate the PARylation results with hits discovered from HTS, we assessed the most potent hit, Compound 4, for its ability to inhibit the viability of SUM149PT tumor organoids. Compound 4 displayed an IC_50_ of 251 μM compared to 85 μM for olaparib at inhibiting organoid viability (Fig. 6C). Although olaparib is a potent inhibitor of PARP1/2 enzyme activity (IC_50_ = 1 nM), this activity only translates to high micromolar cytotoxic activity (IC_50_>50 μM) in cancer cells[21]. Olaparib’s activity in the PARP1–HPF1 complex assay (IC_50_=14 nM) is ~59-fold more potent than compound 4 (~1 μM). Therefore, the difference in cytotoxicity against tumor organoids between olaparib (IC_50_ = 85 μM) and compound 4 (IC_50_ = 251 μM) is consistent with their respective potencies against PARP1-HPF1 complex.

In conclusion, we demonstrate for the first time an HTS assay targeting the HPF1-PARP1 DNA complex. We anticipate this assay will have utility in discovering probes to study the biology of HPF1-PARP1 interactions *in vitro* and in cell models of cancer. Finally, this HTS assay may prove useful in discovering potential lead drugs with efficacy against cancer.

## Supplementary Material

1

2

3
